# *Aesculus hippocastanum* L. Extract Does Not Induce Fibroblast to Myofibroblast Conversion but Increases Extracellular Matrix Production In Vitro Leading to Increased Wound Tensile Strength in Rats

**DOI:** 10.3390/molecules25081917

**Published:** 2020-04-22

**Authors:** Ivan Kováč, Nikola Melegová, Matúš Čoma, Peter Takáč, Katarína Kováčová, Martin Hollý, Ján Ďurkáč, Lukáš Urban, Miriam Gurbáľová, Emil Švajdlenka, Gabriela Mojžišová, Robert Zajíček, Pavol Szabo, Pavel Mučaji, Peter Gál

**Affiliations:** 1 Department of Biomedical Research, East-Slovak Institute of Cardiovascular Diseases, 040 11 Košice, Slovakia; ivankovac.kovi@gmail.com (I.K.); mcoma@vusch.sk (M.Č.); matomeister@gmail.com (M.H.); jan.durkac7@gmail.com (J.Ď.); lurban@vusch.sk (L.U.); 2 Second Department of Surgery, Louise Pasteur University Hospital and Pavol Jozef Šafárik University, 041 90 Košice, Slovakia; 3 Laboratory of Cell Interactions, Center of Clinical and Preclinical Research, MediPark, Pavol Jozef Šafárik University, 040 11 Košice, Slovakia; nikola.melegova5@gmail.com (N.M.); mimagurbalova@gmail.com (M.G.); 4 Department of Pharmacology, Faculty of Medicine, Pavol Jozef Šafárik University, 040 11 Košice, Slovakia; 5 Department of Human and Clinical Pharmacology, University of Veterinary Medicine and Pharmacy, 041 81 Košice, Slovakia; peter.takac@uvlf.sk; 6 Department of Pathology, Louise Pasteur University Hospital, 041 90 Košice, Slovakia; katarina.kovacova@unlp.sk; 7 Department of Chemical Theory of Drugs, Faculty of Pharmacy, Comenius University, 832 32 Bratislava, Slovakia; emilsva@yahoo.com; 8 Department of Natural Drugs, Faculty of Pharmacy, University of Veterinary and Pharmaceutical Sciences, 612 42 Brno, Czech Republic; 9 Department of Experimental Medicine, Faculty of Medicine, Pavol Jozef Šafárik University, 040 11 Košice, Slovakia; gabriela.mojzisova@upjs.sk; 10 Prague Burn Centre, Third Faculty of Medicine and University Hospital Kralovske Vinohrady, Charles University, 100 34 Prague, Czech Republic; robert.zajicek@lf3.cuni.cz; 11 Institute of Anatomy, First Faculty of Medicine, Charles University, 128 00 Prague, Czech Republic; szabopavol@gmail.com; 12 BIOCEV-Biotechnology and Biomedical Centre of The Czech Academy of Sciences and Charles University, First Faculty of Medicine, Charles University, 252 50 Vestec, Czech Republic; 13 Department of Pharmacognosy and Botany, Faculty of Pharmacy, Comenius University, 832 32 Bratislava, Slovakia

**Keywords:** wound healing, repair and regeneration, phytotherapy, horse chestnut

## Abstract

The ability of horse chestnut extract (HCE) to induce contraction force in fibroblasts, a process with remarkable significance in skin repair, motivated us to evaluate its wound healing potential in a series of experiments. In the in vitro study of the ability of human dermal fibroblasts to form myofibroblast-like cells was evaluated at the protein level (Western blot and immunofluorescence). The in vivo study was conducted on male Sprague-Dawley rats with inflicted wounds (one open circular and one sutured incision) on their backs. Rats were topically treated with two tested HCE concentrations (0.1% and 1%) or sterile water. The control group remained untreated. The incisions were processed for wound tensile strength (TS) measurement whereas the open wounds were subjected to histological examination. On the in vitro level the HCE extract induced fibronectin-rich extracellular matrix formation, but did not induced α-smooth muscle actin (SMA) expression in dermal fibroblasts. The animal study revealed that HCE increased wound TS and improved collagen organization. In conclusion, the direct comparison of both basic wound models demonstrated that the healing was significantly increased following HCE, thus this extract may be found useful to improve healing of acute wounds. Nevertheless, the use of an experimental rat model warrants a direct extrapolation to the human clinical situation.

## 1. Introduction

Skin wound healing involves four basic steps: hemostasis, inflammation, proliferation, and maturation [1]. Especially in the proliferation phase, fibroblasts deposit extracellular matrix (ECM) and also produce multiple growth factors, cytokines and chemokines stimulating angiogenesis and supporting the process of re-epithelialization [2,3,4,5,6]. However, many health-impairing conditions result in serious healing-related complications also leading to social and economic issues. Considering economic aspects, therapy based on phytomedicine represents a feasible option of treatment in many regions, thus the request to find new approaches promoting wound repair prompted us to focus on herbal products.

It has been found that *Aesculus hippocastanum* L. extracts (horse chestnut extract–HCE) improve the repair of venous ulcers [7], contraction force in fibroblasts [8], exert antioxidant [9,10] and anti-aging [11] activities. In detail, the generation of contraction force in fibroblasts was associated with the formation of stress fibers and activation of Rho protein and Rho kinase but not by modulating the myosin light chain kinase or other kinases [8]. Furthermore, HCE decreased the expression of MMP-9 and time-dependently increased and decreased the expression of MMP-1 in wounds of streptozotocin-induced diabetic rats [12]. Horse chestnut is a member of the *Hippocastanaceae* family and is distributed worldwide. It has been reported that the HCE extract contains bioflavonoids (quercetin, kaemferol and their diglycosyl derivatives), triterpenoid saponins (escin, prosapogenin), proanthocyanidin A2 and coumarins (esculin and fraxin) [13].

However, only lack of in vivo and/or in vitro data have been published regarding the mechanism of HCE promoting effect on skin wound healing. The exact molecular mechanism underlying the modulation of the fibroblast has particularly been of interest of above mentioned studies. In those papers, [7,8,11,12] addressed critical factors motivated us to perform the current investigation focusing also on other aspects important for skin repair. In particular, the wound tensile strength measurement has been found objective method of wound healing evaluation since a suture may only be removed when the wound is strong enough to withstand the mechanical force during movement [14]. Thus, the present experimental investigation was performed to provide new evidence of the HCE effect on fibroblast functions in skin wound repair on the in vitro and in vivo levels.

## 2. Results

### 2.1. HCE Extract

**Figure 1:**
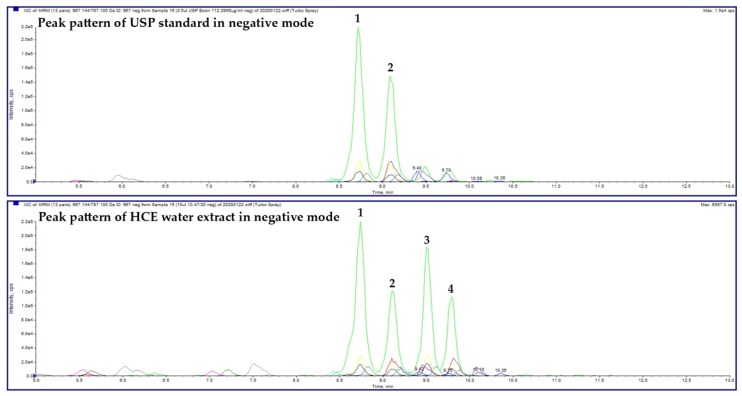
High-performance liquid chromatography (HPLC) analysis of the escin United States Pharmacopeia (USP) Reference Standard (top chromatogram) and horse chestnut water extract (HCE, bottom chromatogram). The main peaks recorded in the negative mode represent escin Ia (1), escin Ib (2), isoescin Ia (3), and isoescin Ib (4).

**Figure 2:**
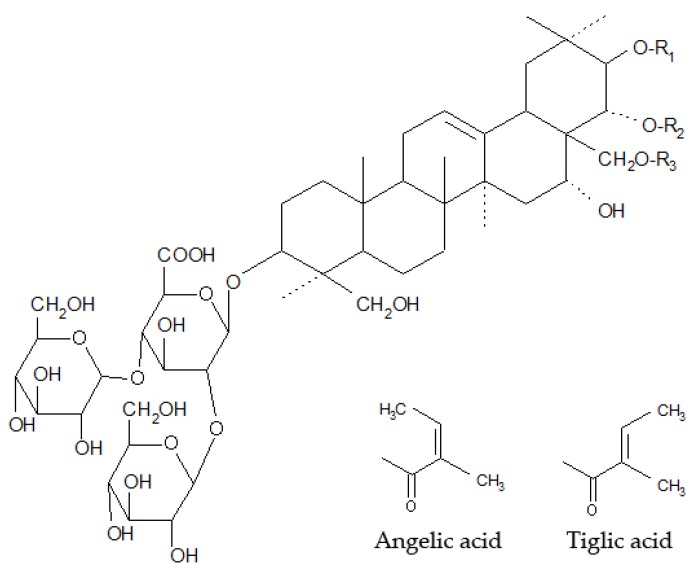
Chemical structures of identified escin isomers (Table 1).

The HPLC analysis revealed that the HCE water extract contains 14.43% of escin isomers. In detail, four main peaks of the extract were recorded in the negative (Figure 1) and positive (not shown) modes of the HPLC. These peaks were recorded with the same retention times as escin Ia, escin Ib, isoescin Ia, and isoescin Ib (Figure 2, Table 1).

### 2.2. In Vitro Study

#### 2.2.1. MTT-Assay of HDFs

**Figure 3:**
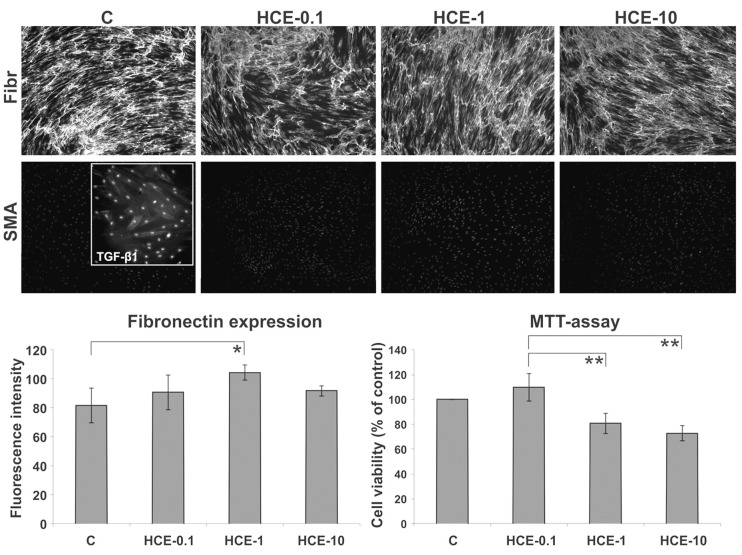
Immunocytochemistry of human dermal fibroblasts (HDFs) demonstrated the ability of HDF to convert into myofibroblasts (see insert, magnification 200×) under the influence of transforming growth factor-β1 (TGF-β1). HCE did not induce transition of fibroblasts into myofibroblast-like cells. The formation of a fibronectin-rich extracellular matrix (ECM) scaffold was most prominent at the HCE concentration of 1 µg/mL whereas the proliferation at the HCE concentration of 0.1 µg/mL (* *p* < 0.05; ** *p* < 0.01).

Firstly, the proliferation activity of cells was examined by means of the MTT-assay. The preliminary experiment included HCE testing at a concentration range from 0.1 to 50 µg/mL (IC50 = 18 μg/mL). Then, the main experiment was conducted bellow the IC50 concentration (0.1, 1 and 10 μg/mL) where only the lowest tested concentration of HCE increased the metabolic activity of cells, whereas both higher tested concentrations were found inhibitory to cell proliferation when compared to the control (Figure 3).

#### 2.2.2. Western Blot Analysis of HDF

**Figure 4:**
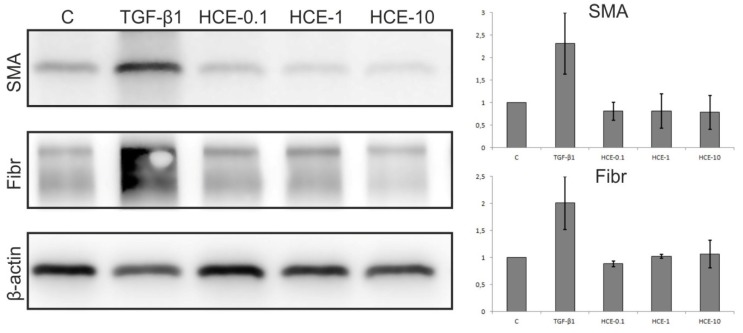
Western blot analysis of human dermal fibroblasts (HDF) revealed that medium containing TGF-β1 induced expression of α-smooth muscle actin (SMA) and fibronectin (Fibr) whereas cells treated with horse chestnut water extract (HCE) expressed slightly lower levels of SMA with no concentration dependence.

Results from the Western blot (WB) analysis are summarized in Figure 4. Medium containing TGF-β1 was used as positive control to induce the expressions of α-smooth muscle actin (SMA) and fibronectin. HDFs treated with HCE expressed slightly lower levels of SMA with only weak concentration dependence. In this line of evidence densitometric analysis supported insignificant poor down-regulation of SMA expression with increasing HCE concentration. Intriguingly, HCE did not affect intracellular levels of fibronectin despite its high extracellular deposition revealed by immunofluorescece analysis. 

#### 2.2.3. ICC Analysis of HDFs

Cell treatment with the HCE led to the deposition of a fibronectin-rich ECM scaffold (Figure 3). Interestingly, the most prominent newly synthesized ECM network was observed on the coverslips with cells exposed to the extract concentration of 1 µg/mL where cells revealed lower proliferation activity when compared to that of a control and 0.1 µg/mL of tested HCE. 

The ability to form myofibroblasts was tested by the addition of TGF-β1 (positive control, see insert in Figure 3) into the culture medium and was confirmed by the presence of SMA expressing myofibroblast-like cells. However, the presence of HCE in the culture medium did not stimulate any conversion of fibroblasts into myofibroblast-like cells (Figure 3). 

### 2.3. In Vivo Animal Study

#### 2.3.1. Wound Tensile Strength

**Figure 5:**
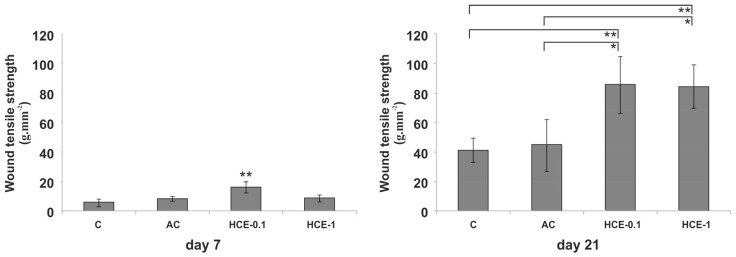
Wound tensile strengths (all data are presented as mean ± SD) of wounds removed from untreated control (C), aqueous control (AC), HCE-0.1% treated, and HCE-1% treated groups at day 7 and 21 post-surgery (* *p* < 0.05; ** *p* < 0.01).

Results from the wound TS measurement at day 7 and 21 are shown in Figure 5. At day 7 post wounding the only significantly increased wound TS was found in the HCE-0.1 group (HCE-0.1 = 16.4 ± 3.8 g/mm^2^). No differences were seen between control (C = 5.9 ± 2.5 g/mm^2^), aqueous control (AC = 8.5 ± 1.7 g/mm^2^) and HCE-1 (HCE-1 = 8.9 ± 2.5 g/mm^2^) groups (Figure 5, left graph). 

On the other hand, at day 21 skin wound treatment with both studied concentrations of HCE (HCE-0.1 and HCE-1) significantly increased tensile strength (TS) in comparison with control group (C = 41.4 ± 8.4 g/mm^2^) and aqueous control group (AC = 44.7 ± 17.4 g/mm^2^). Of note, the differences between both HCE-treated groups (HCE-0.1 = 85.9 ± 19.2 g/mm^2^; HCE-1 = 84.5 ± 14.8 g/mm^2^) were not found significant (Figure 5, right graph).

#### 2.3.2. Histology 

**Figure 6:**
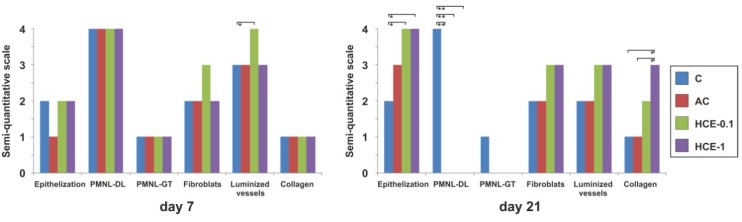
Semi-quantitative analysis of histological structures and processes evaluated at day 7 and 21 post-surgery. Data are presented as median (* *p* < 0.05; ** *p* < 0.01).

The data from the semi-quantitative scoring of histological assessment at day 7 and 21 are shown in Figure 6.

**Figure 7:**
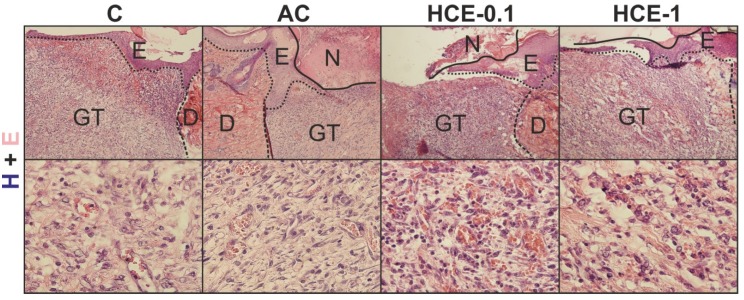
Figures show skin wounds removed at day 7 after surgery. Hematoxylin and eosin (H + E) staining is shown in the first (magnification 100×) and second (magnification 600×) horizontal panel. The most prominent presence of luminized vessels may be seen in the HCE-0.1% group.

Although at day 7 the acute inflammatory reaction was finished, wounds (Figure 7) were not yet completely bridged by a new epithelial layer with no remarkable differences between groups. All wounds were characterized by well-formed granulation tissues. The most interesting difference was seen in the HCE-0.1 group where wounds displayed increased presence of luminized vessels when compared to all other groups. Based on the Van Gieson staining no remarkable differences were seen in the presence of collagen (not shown).

**Figure 8:**
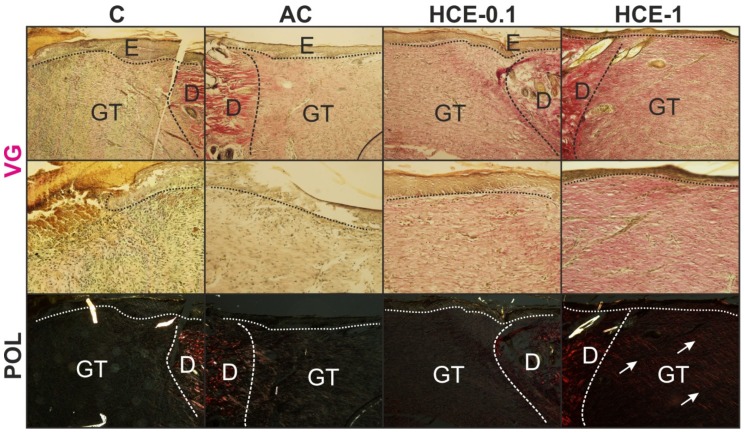
Figures show skin wounds removed at day 21 after surgery. Van Gieson staining is shown in the first (magnification 100×) and second (magnification 200×) horizontal panel. Well formed GT rich on luminized vessels and fibroblasts can be observed in both HCE-treated groups (HCE-0.1 and HCE-1). Collagen type-1 fibers are visualized by means of the polarized light in the second horizontal panel. The most prominent organization of collagen into fibers can be seen in the HCE-1% treated wounds (white arrows).

Twenty-one days following surgery (Figure 8), regeneration of the epidermis was completely finished in the AC group, both HCE groups, but not in the untreated control (C). As a result of incomplete re-epithelialization the demarcation line was still present and the superficial parts of the granulation tissue (GT) were slightly infiltrated with inflammatory cells. In this period remodeling and reorganization of the GT dominated, thus the scar was created. Only a mild number of luminized vessels were present in the GT tissues of wounds with no remarkable differences between groups. Both HCE groups had slightly increased numbers of fibroblasts and luminized vessels when compared to other groups, resulting also in significantly improved deposition and organization of collagen fibers as seen under the polarized light; however, only in HCE-1 group.

## 3. Discussion

In the present study, we demonstrated for the first time that HCE significantly increased wound tensile strength in rats. It is known that the wound stiffness is reflected by the composition of the newly formed ECM in the incisional gap [15]. On the in vitro level, we also observed the most prominent ECM production by dermal fibroblasts following HCE treatment. The ECM proteins are produced and organized by fibroblasts, one of the main components of the GT [1]. While increased deposition of collagen was recorded in both treated groups its organization into polarized-light reflecting fibers was rather seen in the group treated with the higher tested HCE concentration. This result was also confirmed by wound TS measurement to address the clinical significance of present study. However, the increased wound TS in HCE-treated wounds may also be attributed to the escin-mediated anti-inflammatory properties [16]. Therefore, the missing data on the early phases of wound healing might present the first limitation of our current investigation. Although we previously observed differences in the efficiency of treatment between sutured incisions and open excisions [17,18], the HCE treatment resulted in observable healing improvement in both basic wound models.

Our extract contained four main components recognized as the escin Ia, escin Ib, isoescin Ia, and isoescin Ib [19]. The biologically active and dominant compound of the HCE, β-escin, primarily composed of escin Ia and escin Ib [13], was not identified by others as the active component inducing cell force [8,11]. Furthermore, its oral administration did not accelerate bone fracture healing of tibia or modulated hydroxyproline levels in the abdominal incision wounds in rabbits [20]. However, it is rather unlikely that the ballast compounds may be found responsible for the HCE biological and/or healing-promoting activities. An observation also supported by our in vitro study where HCE in the absence of TGF-β1 did not modulate SMA expression in fibroblasts. Of note, the TGF-β1-induced SMA expression was rather decreased following HCE treatment (unpublished data) that may eventually support the anti-cancer properties of escin [21] and should also be examined in further studies conducted on cancer-associated fibroblasts. In this context missing data on direct interactions between the TGF-β1 cytokine and its receptor on the one side and escin on the other side present the second limitation of our current study.

On the other hand, the potential mechanism of HCE may also be attributed to its anti-oxidant activities [10]. That study also revealed that the whole extract exerted better anti-radical properties compared to β-escin, the dominant active ingredient of the HCE. In particular the mixture of flavonoids may act as an effective free radical-scavenging system possibly also working as a wound healing accelerator [22]. However, the extraction technique used in the preparation of our extract did not resulted in the presence of flavonoids. Therefore, it is an open question for further research to what extent does the mixture of the four identified escin isomers have the capability to modulate the course of skin wound healing.

## 4. Materials and Methods 

### 4.1. Plant Material and Preparation of the Aqueous Extract

Horse chestnut (*Aesculus hippocastanum* L.) water extract (HCE) (Calendula a.s., Nová Ľubovňa, Slovakia) and was provided in the form of a dry powder.

### 4.2. HCE Extract Analysis

Qualitative and quantitative analysis of HCE sample was realized by HPLC-DAD-MS according to previously reported method [23]. Briefly, all HPLC analysis were carried out on the Agilent Technologies 1200 Series chromatograph (Agilent Technologies Deutschland, Walbron, Germany), equipped with a diode array spectrometer and MS ion trap detectors (AB SCIEX 3200 Q TRAP LC/MS/MS System, Applied Biosystems, Foster City, CA, USA). MS spectra were recorded in negative and positive mode in the range of 1065-1175 Da. An Agilent (Santa Clara, CA, USA) column Poroshell 120 EC-C18 (4.6 × 100 mm, 2.7-Micron, P.N. 695975-902) was used as stationary phase. The mobile phases were solvent A (methanol with 1 mM HCOONH4 and 1% HCOOH) and solvent B (H_2_O with 1 mM HCOONH4 and 1% HCOOH). The elution gradient: 0 min 70% A + 30% B; 7 min. 100% A; 13 min. 100% A; 13.1 min. 70% A + 30% B; 20 min. 70% A + 30% B. Flow: 0.3 mL/min, temperature: 30 °C. Constituents’ identification was obtained on the basis of MSn fragmentation experiments and by comparison of the fragmentation pathways with reference compound of β-escin (USP Reference Standard, 99% purity, Merck, Taufkirchen, Germany), batch no. R023G0. Quantification of saponins in the sample was realized according to the calibration curve (data not shown) of β-escin (USP) at six different concentrations.

### 4.3. In Vitro Experiments

#### 4.3.1. Primary Cultures of Human Dermal Fibroblasts (HDFs) 

Human dermal fibroblasts (HDFs) were isolated from residual skin samples obtained during reconstructive surgery with informed consent of the patient (in agreement with the Helsinki Declaration after approval by the Ethical Committee of the Third Faculty of Medicine) at the Prague Burn Centre (Charles University, Third Faculty of Medicine and University Hospital Kralovske Vinohrady). Briefly, small pieces of split-thickness skin grafts were enzymatically treated with 0.25% trypsin (Sigma-Aldrich, St. Louis, MO, USA) at room temperature for 15 min. to separate the epidermis from the dermis. The dermis was then cut into small pieces that were seeded on cultivation dishes and covered with Dulbecco′s medium (DMEM) supplemented with 10% fetal bovine serum (FBS) and antibiotics (penicillin and streptomycin, Biochrom, Berlin, Germany) [24]. After a few days migrating cells were trypsinized and expanded by further culturing at 37 °C and 5% CO_2_. Cells at passages 7–8 were used in all experiments. 

#### 4.3.2. MTT-Assay

Cells were seeded (5000 cells/100 µL/well) into 96-well-plates in culture medium (10% FBS). Twenty-four h after seeding the medium was changed to medium with the presence (0.1, 1, and 10 µg/mL final concentration whereas the final concentrations of DMSO did not exceed 0.1%) or absence of HCE. Following the next 48 h of incubation, the MTT salt (0.45 mg/mL final concentration) was added and cells were incubated for another 3 h. After the medium with MTT was removed, DMSO was added to lyse the cells. Subsequently, the absorbance at λ = 570 nm was determined with the Infinite M200 spectrofluorometer (Tecan, Männedorf, Switzerland). The amount of created formazan (correlating to the number of viable and metabolically active cells) was calculated as a percentage of control cells.

#### 4.3.3. Western Blot (WB) of HDFs

Protein lysates were obtained from HDFs seeded at passage six on Petri dishes at the density of 5000 cells/cm^2^ and cultivated for seven days in the presence (0.1, 1, and 10 µg/mL) or absence (control) of tested HCE extract. TGF-β1 at final concentration of 30 ng/mL of (PeproTech, London, UK) was used as positive control of fibroblast to myofibroblast differentiation [25,26]. The set of primary and secondary antibodies applied in the analysis is shown in Table 2. Briefly, cells were washed with cold PBS (phosphate-buffered saline) and collected in Laemmli sample buffer (100 mM TRIS-HCL pH approx. 6.8, 10% glycerol, 2% SDS) containing protease and phosphatase inhibitors (Sigma-Aldrich). Immediately after collection, cells were disrupted using sonicator (QSonica, 40% amplitude, 15 s). After boiling (95 °C, 5 min), samples were separated in SDS-PAGE gel (10% Bis-Tris) and transferred to PVDF membrane using iBlot 2 (Thermo Fischer Scientific). Following 1 h of blocking in 5% NFDM/BSA (non-fat dry milk/bovine serum albumin) dissolved in TBS (tris-buffered saline) with 0.1% Tween at room temperature, membranes were incubated with the primary antibody at 4 °C overnight. The next day, membranes were incubated with HRP-conjugated secondary antibodies for 1 h at room temperature. Protein bands were detected using ECL (SuperSignal West Pico PLUS chemiluminescent Substrate, Thermo Fischer Scientific) and signal was acquired at MF-ChemiBis 2.0 (DNR Bio-Imaging Systems). β-actin was used as sample loading control. Chemiluminescent signal of all detected proteins was quantified using the Image Studio (LI-COR) western blot densitometry software and normalized to β-actin.

#### 4.3.4. Immunocytochemistry (ICC) of HDFs

HDFs were seeded at a density of 3000 cells/cm^2^ and cultivated for 10 days in the presence (0.1, 1, and 10 µg/mL) or absence (control) of tested HCE. Medium containing 30 ng/mL of TGF-β1 (PeproTech) was used as positive control of whether used primoculture of fibroblasts is able to convert into myofibroblast-like cells. Briefly, cells were fixed with 2% buffered paraformaldehyde (pH 7.2) for 5 min. and washed with PBS. Cell membranes were permeabilized by Triton X-100 (Sigma-Aldrich) and sites for the antigen-independent binding of antibodies were blocked by porcine serum albumin (DAKO, Glostrup, Denmark). Commercial antibodies were diluted according to manufacturer’s instructions. The set of used antibodies for the immunofluorescent analysis is shown in Table 2. The specificity of the immunocytochemical staining was controlled by replacement of the first-step antibody by an irrelevant antibody and by testing of positive control samples. Cell nuclei were stained by 4′,6-diamidino-2-phenylindole (DAPI; Sigma-Aldrich). All samples were mounted in Vectashield (Vector Laboratories, Burlingame, CA, USA) and inspected by the Eclipse 90i fluorescence microscope (Nikon, Tokyo, Japan) equipped with filter cubes for FITC, TRITC, DAPI and Cool-1300Q CCD camera (Vosskühler, Osnabrück, Germany). Images were recorded and analyzed by the LUCIA 5.1 software (Laboratory Imaging, Prague, Czech Republic).

### 4.4. In Vivo Experiment

#### 4.4.1. Animal Model

The experiment was approved on July 28th 2015 by the Ethical committee of the Faculty of Pharmacy at Comenius University and by the State Veterinary and Food Administration of the Slovakia (Ro-2617/15-221b).

Male Sprague-Dawley rats (*n* = 56), weighing 400 ± 40 g, were obtained from the Animal Facility of P. J. Šafárik University and used for experiment. Animals were individually housed under standard conditions (55 ± 5% humidity, 22 ± 2 °C, 12/12 h light-dark cycle) in plexiglass cages with free access to standard laboratory diet and tap water.

**Figure 9:**
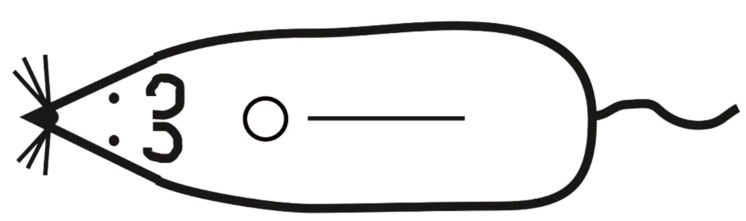
Positions of the circular excision (1 cm in diameter) and the longitudinal sutured incision (4 cm in length) on the back of a rat.

Based on treatment rats were randomized into four groups. In general anesthesia (33 mg/kg of ketamine i.m. [Calypsol, Richter Gedeon, Budapest, Hungary], 11 mg/kg i.m. of xylazine (Rometar a.u.v., Spofa, Prague, Czech Republic), and 5 mg/kg of tramadol (Tramadol-K, Krka d.d., Novo Mesto, Slovenia) and under aseptic conditions one 4 cm long full-thickness skin incision and one round (1 cm in diameter) full thickness skin excisions were inflicted on the dorsum of all rats (Figure 9). The incision was closed using intradermal running suture (Chiraflon 5/0, Chirmax, Prague, Czech Republic). Following surgery wounds remained undressed and all animals were housed individually to avoid wound damage. Half of animals from each group were killed by an overdose of anesthetics at day 7 and the other half at day 21 post wounding. 

#### 4.4.2. Wound Treatment

For wound treatment the solution was prepared by diluting the dried extract in sterile water. In this experiment two HCE concentrations were tested: HCE-1–1g of lyophilized extract diluted in 100 mL of sterile water and a 10-times diluted concentration–HCE-0.1. Consecutively, the extract was filtered (0.2 μm).

The control group (C, *n* = 14) remained untreated. To exclude the effect of moist healing wounds were in parallel topically treated with sterile water (AC, *n* = 14). Similarly, in HCE-treated groups the extract of either higher (HCE-1, *n* = 14) or lower (HCE-0.1, *n* = 14) concentration was topically applied to the wound surface. Each treatment was performed by an eye dropper three times a day during the first three days following wounding. To assure equal stress for all rats the control group was shame treated.

#### 4.4.3. Wound Tensile Strength Measurement

The tensile strength (TS) testing device was developed in our laboratory. Briefly, it is based on a specially shaped horizontal arm pulling one side of a sample with the opposite side fixed to a measuring tip of a force meter unit (OMEGA Engineering, Inc., Stamford, CT, USA). The moving arm is driven by a high-precision stepper motor MDI-17 (Intelligent Motion Systems, Inc., Marlborough, CT, USA) through a linear slider.

The measurement technique was described previously [27]. Briefly, two 1-cm-wide skin strips were removed from each incision and placed lengthwise between the clamps of the TS testing device. Pulling was performed perpendicularly to the original direction of the wound. The maximal breaking strength was recorded for each sample. The TS was then calculated by using the formula: TS = MRS/A (MBS = maximal rupture strength [g], A = wound area [mm^2^]) and expressed in g/mm^2^.

#### 4.4.4. Basic Histology and Semi-quantitative Scoring of Wounds

Wound specimens were routinely processed for light microscopy (fixation in 4% buffered formaldehyde, dehydration using increasing concentration of alcohol, paraffin embedding, sectioning (5 µm thick), and staining with hematoxylin-eosin (HE)).

Used semi-quantitative method was described previously [28]. We evaluated, in a blinded fashion, the re-epithelialization of the epidermis, the presence of inflammatory cells (polymorphonuclear leukocytes [PMNLs], fibroblasts, luminized vessels, and new collagen [also by polarized light]). The examined structures/processes were scored from 0 to 4 (Table 3).

### 4.5. Statistical Analysis

The wound tensile strengths are expressed as mean ± standard deviation (SD) and were compared by one-way ANOVA, followed by Tukey–Kramer multiple comparison test. The non-parametric data from the semi-quantitative analysis are expressed as median and were compared by Kruskal–Wallis test. Level of *p* < 0.05 was considered to be statistically significant. 

## 5. Conclusions

In conclusion, the direct comparison of both basic wound models demonstrated that the healing was significantly increased following HCE, thus this extract may be found useful in improving acute skin wound healing. However, we did not observe a direct effect of HCE on fibroblasts to myofibroblast transition, and thus it is essential to perform further experiments aimed at finding the exact underlying mechanism of action and optimal therapeutic protocol. Finally, a direct extrapolation from this experimental model to the human clinical situation presents the third limitation of our study due to inter-species variability. However, general molecular regulation of wound healing should be similar, and thus the present data encourages further respective investigations in other animal models physiologically and evolutionary closer to humans.

## Figures and Tables

**Table 1:** Chemical structure of escin isomers (explanation to Figure 3).

Saponin	R_1_	R_2_	R_3_
Escin Ia	Tiglic acid	-COCH_3_	H
Escin Ib	Angelic acid	-COCH_3_	H
Isoescin Ia	Tiglic acid	H	-COCH_3_
Isoescin Ib	Angelic acid	H	-COCH_3_

**Table 2:** Reagents used for western blot and immunofluorescence.

**Antibodies Used for Western Blot**						
**Primary Antibody**		**Abbreviation**	**Host**	**Isotype**	**Clonality**	**Produced by**
α-smooth muscle actin		SMA	rabbit	IgG	monoclonal	CST, USA
Fibronectin		Fibr	rabbit	IgG	monoclonal	Abcam, UK
β-actin		β-actin	rabbit	IgG	monoclonal	CST, USA
**Secondary Antibody**		**Abbreviation**	**Host**	**Isotype**	**Clonal**	**Produced by**
Anti-rabbit, HRP-linked			goat	IgG		CST, USA
**Antibodies Used for Immunofluorescence**						
**Primary Antibody**	**Abbreviation**	**Host**	**Produced by**	**Secondary Antibody**	**Produced by**	**Channel**
α-smooth muscle actin	SMA	mouse monoclonal	DakoCytomation, Glostrup, Denmark	goat anti-mouse	Sigma-Aldrich, St. Louis, MO, USA	TRITC-red
Vimentin	Vim	mouse monoclonal	DakoCytomation, Glostrup, Denmark	goat anti-mouse	Sigma-Aldrich, St. Louis, MO, USA	TRITC-red
Fibronectin	Fibr	rabbit polyclonal	DakoCytomation, Glostrup, Denmark	swine anti-rabbit	Biotechnology, Santa Cruz, CA, USA	FITC-green

**Table 3:** Explanation of used scale of the semi-quantitative analysis of histological sections (ST—surrounding tissue, i.e., tissue out of GT; DL—demarcation line; SCT—subcutaneous tissue; GT—granulation tissue).

Scale	Epithelialization	PMNL	Fibroblasts	Luminized Vessels	Collagen
0	thickness of cut edges	absent	absent	absent	absent
1	migration of cells (<50%)	mild ST	mild-ST	mild-SCT	minimal-GT
2	migration of cells (≥50%)	mild DL/GT	mild-GT	mild-GT	mild-GT
3	bridging the excision	moderate DL/GT	moderate-GT	moderate-GT	moderate-GT
4	keratinization	marked DL/GT	marked-GT	marked-GT	marked-GT
